# Supplementary figures and images for: SARS-CoV-2 transmission among health care workers, an outbreak investigation using whole-genome sequencing
Source: PLoS One. 2023 Mar 31;18(3):e0283292. doi: 10.1371/journal.pone.0283292 (PMC10065229; doi:10.1371/journal.pone.0283292)

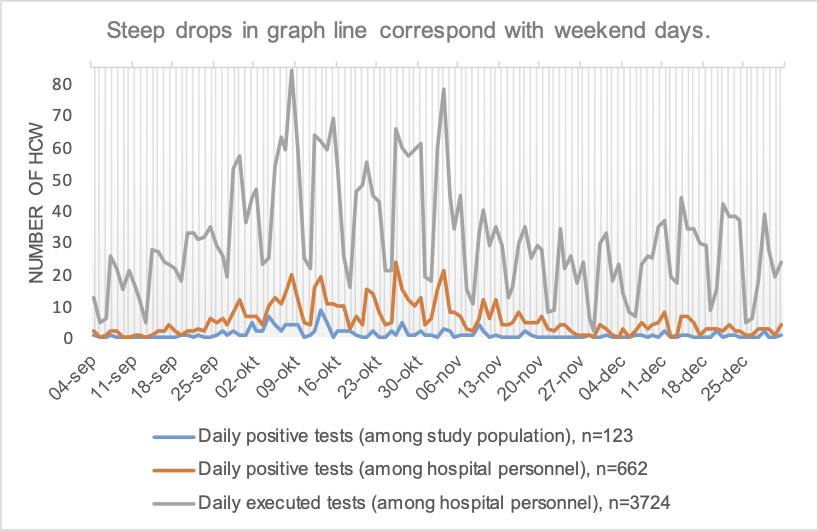

Supplement: S1 Fig — (TIF) [file pone.0283292.s001.tif]

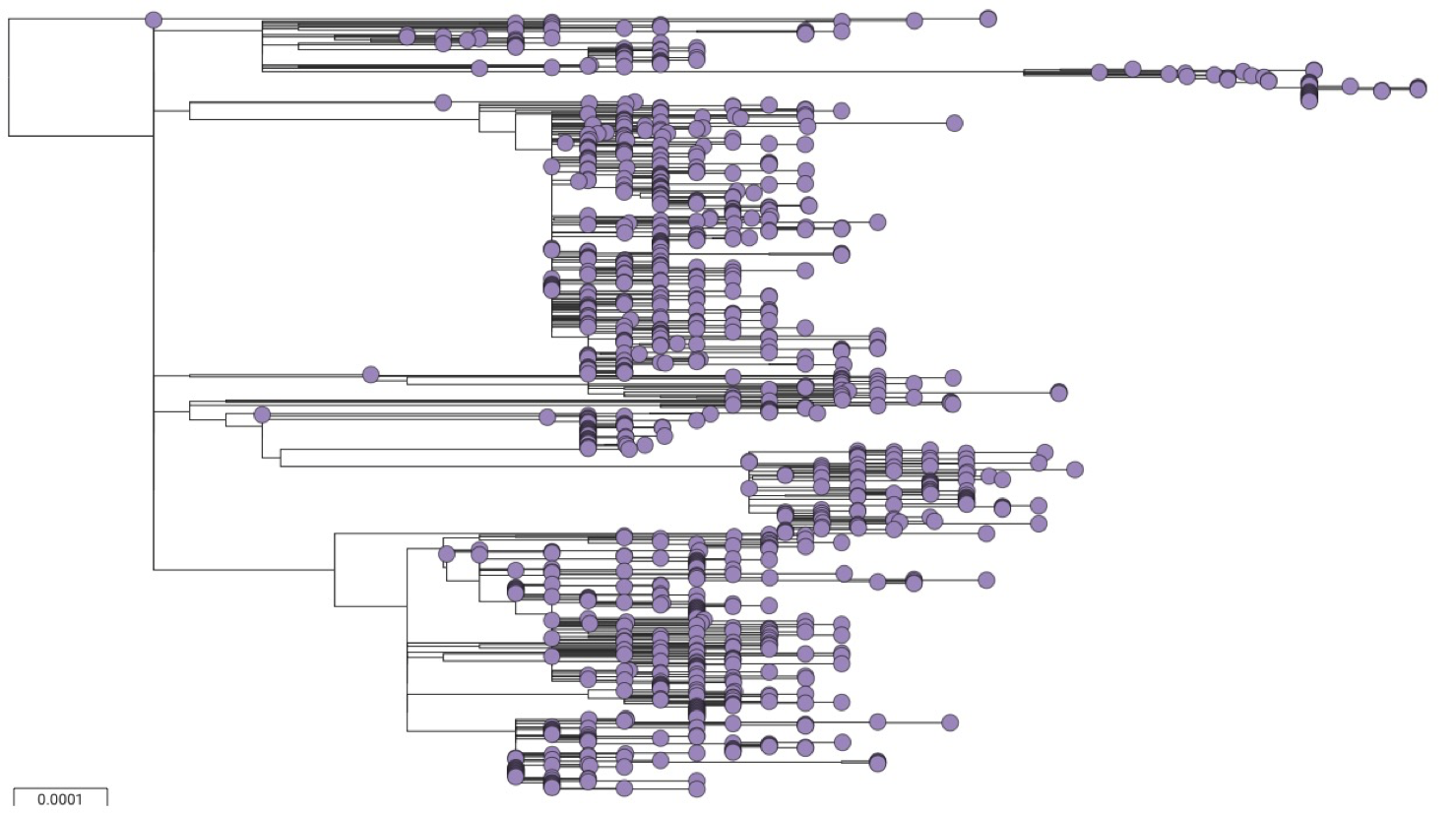

Supplement: S2 Fig — This figure depicts the total phylogenetic tree with the study population and regional sequences combined. Regional sequences of the concerning province are downloaded from GISAID. (TIF) [file pone.0283292.s002.tif]

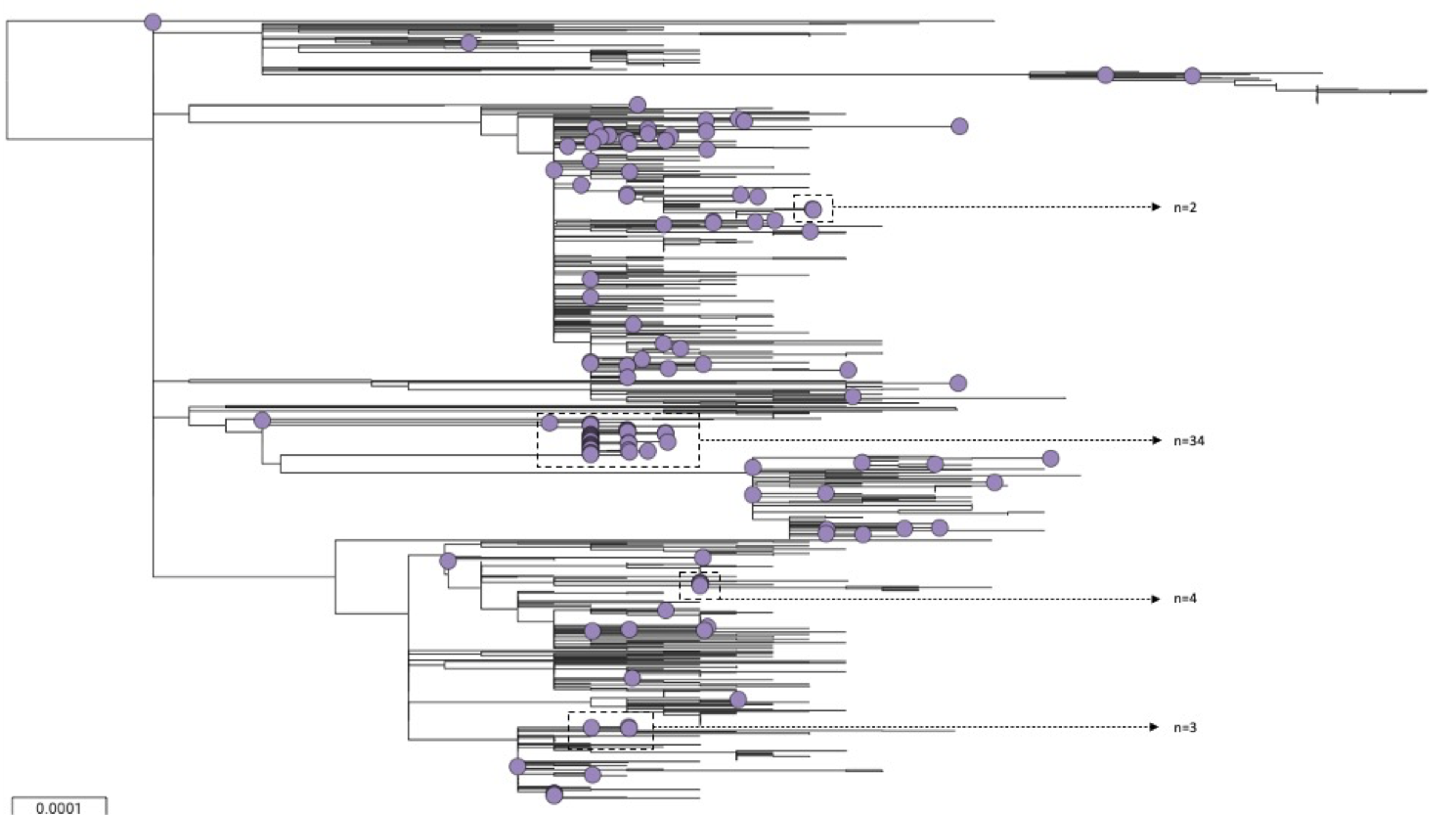

Supplement: S3 Fig — The purple circles represent the study population. The four clusters are indicated. Tree branches without purple circles represent filtered out regional sequences. Regional sequences of the concerning province are downloaded from GISAID. (TIF) [file pone.0283292.s003.tif]

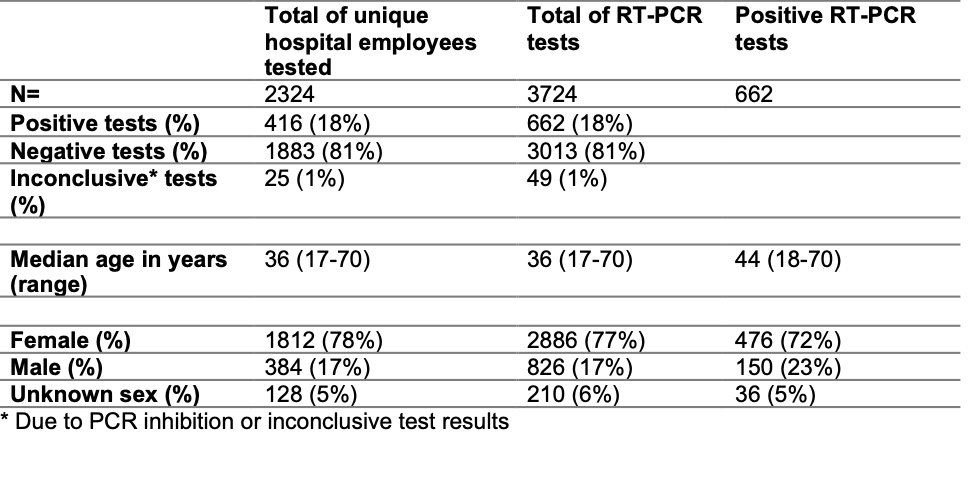

Supplement: S1 Table — (TIFF) [file pone.0283292.s004.tiff]
